# Role of ambient temperature in modulation of behavior of vanadium dioxide volatile memristors and oscillators for neuromorphic applications

**DOI:** 10.1038/s41598-022-23629-4

**Published:** 2022-11-12

**Authors:** Stefania Carapezzi, Corentin Delacour, Andrew Plews, Ahmed Nejim, Siegfried Karg, Aida Todri-Sanial

**Affiliations:** 1 Microelectronics Department, LIRMM, University of Montpellier, CNRS, Montpellier, 34095 France; 2 Department of Electrical Engineering, Eindhoven University of Technology, Eindhoven, 5612 AP The Netherlands; 3 Silvaco Europe Ltd., St Ives, PE27 5JL UK; 4 Department of Science and Technology, IBM Research Europe - Zurich, Ruschlikon, 8803 Switzerland

**Keywords:** Electronic devices, Electronic devices

## Abstract

Volatile memristors are versatile devices whose operating mechanism is based on an abrupt and volatile change of resistivity. This switching between high and low resistance states is at the base of cutting edge technological implementations such as neural/synaptic devices or random number generators. A detailed understanding of this operating mechanisms is essential prerequisite to exploit the full potentiality of volatile memristors. In this respect, multi-physics device simulations provide a powerful tool to single out material properties and device features that are the keys to achieve desired behaviors. In this paper, we perform 3D electrothermal simulations of volatile memristors based on vanadium dioxide (VO$_{2}$) to accurately investigate the interplay among Joule effect, heat dissipation and the external temperature $T_{0}$ over their resistive switching mechanism. In particular, we extract from our simulations a simplified model for the effect of $T_{0}$ over the negative differential resistance (NDR) region of such devices. The NDR of VO$_{2}$ devices is pivotal for building VO$_{2}$ oscillators, which have been recently shown to be essential elements of oscillatory neural networks (ONNs). ONNs are innovative neuromorphic circuits that harness oscillators’ phases to compute. Our simulations quantify the impact of $T_{0}$ over figures of merit of VO$_{2}$ oscillator, such as frequency, voltage amplitude and average power per cycle. Our findings shed light over the interlinked thermal and electrical behavior of VO$_{2}$ volatile memristors and oscillators, and provide a roadmap for the development of ONN technology.

## Introduction

The Internet of Things (IoTs)—one of the leading technologies for the twenty first century—presents an unprecedented opportunity to provide ubiquitous computing, sensing and learning capabilities at the edge. Many smart applications in key fields such as healthcare, transport, autonomous cars, or smart cities, to name a few, are fast adopting IoTs with embedded artificial intelligence (AI) on them to perform a diverse set of applications. Presently, most of the deployed AI on edge devices are pre-trained or send data to the cloud or data centers^1^. But with the continuous increase in edge devices and the need for real-time data processing, such a procedure is becoming unsuitable due to data latency, privacy, bandwidth saturation, and power consumption^2^. Alternatively, an efficient way is to train AI algorithms on IoT devices by handling data locally where they are amassed. However, executing AI algorithms on current computing architecture based on von Neumann one suffers from large power consumption, mainly due to energy loss of data transfer between memory and processor. This clashes with the limited power and computing resources available on the edge. Therefore, novel concepts are required to go beyond von Neumann computing era to enable AI learning and inference at the edge.

**Figure 1:**
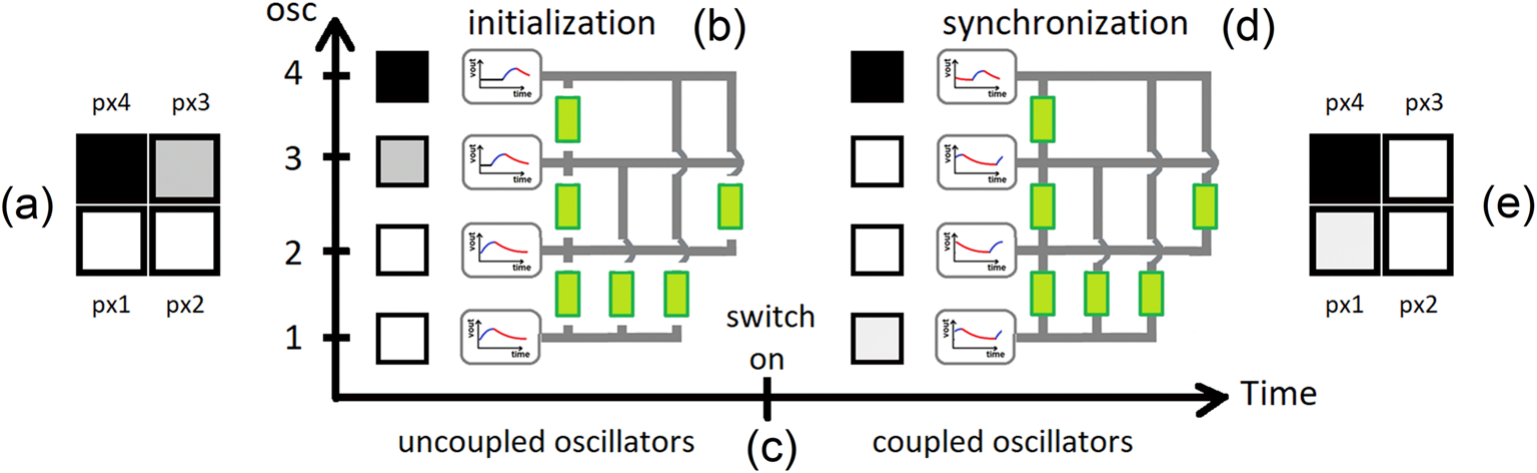
Example of possible ONN application for image recognition tasks. (**a**) Input image. (**b**) Initialization of ONN circuit. Each pixel of input image is mapped as oscillator’s phase $\Delta \Phi _{\mathrm{in, j}}$ by delaying of $\Delta t_{\mathrm{in, j}}$ the biasing $V_{\mathrm{DD, j}}$ compared to the reference (bottom-most) oscillator. The computation begins at coupling of all the oscillators (**c**), where the simplest coupling elements are resistances $R_{\mathrm{C, i,j}}$ (green blocks). (**d**) The oscillators synchronize in a stable in-phase ($\Delta \Phi _{\mathrm{out, j}} = 0^{\circ }$) or out-of-phase ($\Delta \Phi _{\mathrm{out, j}} \ne 0^{\circ }$) state. The phase of each oscillator is mapped back as pixel (gray scale) color to yield the (**e**) output image.

Both academia and industry are exploring beyond von Neumann architectures, aiming to compute by merging memory and processing of information. While many alternatives are being scrutinized, analog computing based on the principle of “let physics compute” presents a quite interesting solution^3^. At its core, it transforms the dynamics of complex systems such as coupled oscillatory neural networks (ONNs)^2,3^ into an energy-efficient computing tool. ONNs take inspiration from the human brain, which is able to perform sophisticated tasks with a reduced energy budget. Specifically, ONNs aim to emulate biological neural networks, which are made of elemental units outputting periodic signals (the neurons) and interconnected into systems (the networks)^4–8^. ONN circuits mimic neural nets as ensembles of coupled oscillators (Fig. 1), where the information is encoded in the phase difference among the oscillators (phase-based computing).

To compute with ONNs, oscillators must be initialized with different delay times compared to a reference oscillator (Fig. 1b). This establishes an initial phase difference between each oscillator and the reference one. Then, at a given time, all oscillators are connected to each other through synaptic coupling elements (Fig. 1c), where the simplest coupling elements are resistors or capacitors. These couplings mediate the ONN interaction and allow to achieve a collective phase synchronization, if any, which is the result of ONN computing (Fig. 1d)^9^.

An example of application on ONN is pattern recognition, given that the phase state of each oscillator can be mapped to a white ($\Delta \Phi _{\mathrm{j}} = 0^{\circ }$), a black ($\Delta \Phi _{\mathrm{j}} = 180^{\circ }$) or a gray (all other values of $\Delta \Phi _{\mathrm{j}}$) pixel (Fig. 1a,e). Overall, ONNs have attracted immense interest because they have a simple structure yet fast computation and energy efficient computing paradigm^10–13^.

**Figure 2:**
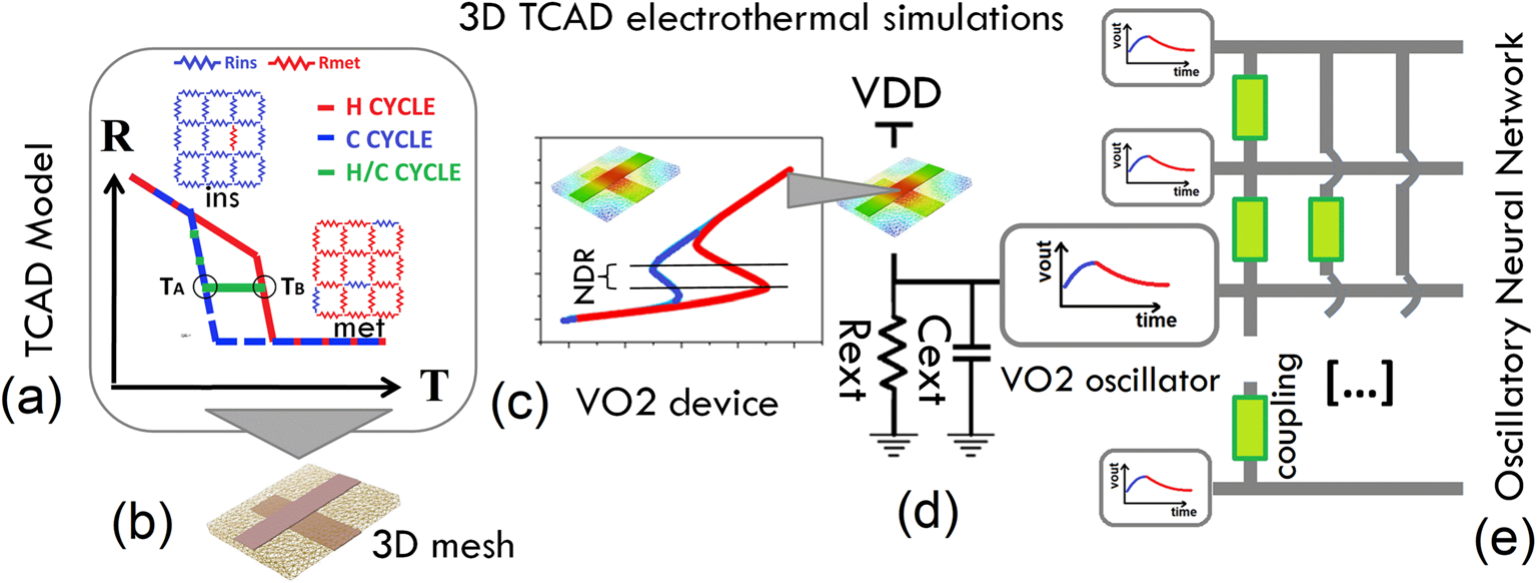
(**a**) Temperature-triggered resistive switching of VO$_{2}$ volatile memristor. (**b**) 3D mesh of VO$_{2}$ CB device. (**c**) TCAD simulated *I*–$V_{\mathrm{D}}$ characteristics. The associated NDR region has been highlighted. (**d**) Scheme of VO$_{2}$ oscillator circuit. Self-oscillatory electrical behavior is associated to a suitable choice of the load-line. (**e**) ONN.

Oscillators are the basic building blocks of ONNs. In this paper, we report on the realization of beyond CMOS oscillators based on vanadium dioxide (VO$_{2}$) volatile memristors. Volatile memristors are currently widely investigated for applications as neural/synaptic devices, or as random generators for hardware security^14,15^. Their operation mode relies on a mechanism of volatile resistive switching. VO$_{2}$ is a Transition Metal Oxide that changes from a high-resistive monoclinic (M$_1$) crystal structure to a low-resistive tetragonal rutile-like (R) structure. Such abrupt resistive switching is related to temperature overcoming a threshold (340 K) close to room temperature. This process takes the name of insulator-to-metal transition (IMT), it is reversible (metal-to-insulator transition, MIT) and often hysteretic (Fig. 2a). The overall variation of VO$_{2}$ resistivity across IMT/MIT can be up to several orders of magnitude.

The resistive switching of VO$_{2}$ has been experimentally observed also in channels of two-terminal VO$_{2}$ devices due to current injection or voltage biasing. This gives rise to the so called volatile Mott memristors^14–18^ because, upon removal of electrical stimulus, these devices reset back spontaneously to the high resistive state (HRS) from the low resistive state (LRS). Experimental evidence^19–22^ points towards self-heating as driving this transition by increasing the device temperature.

A negative differential resistance (NDR) region in the current-voltage (I–V) characteristics (Fig. 2c) of VO$_{2}$ volatile memristors is the hallmark of such resistive switching. This provides the possibility to build VO$_{2}$ oscillators by simply inserting a VO$_{2}$ volatile memristor into an RC circuit (Fig. 2d)^17,22,23^, without need for inductive components. The oscillatory regime is achieved provided that the circuit load-line $I_{L} = (V_{\mathrm{DD}} - V) / R_{\mathrm{ext}}$ crosses the NDR region of current *I* vs. voltage across the VO$_{2}$ ($V_{\mathrm{D}}$) curve, where $V_{\mathrm{DD}}$ is the applied bias and $R_{\mathrm{ext}}$ is the external resistance. Thus, the periodic oscillation is due to cyclic charging and discharging of external capacitor $C_{\mathrm{ext}}$ related to VO$_{2}$ device being in LRS and HRS (circuit topology of Fig. 2d).

Due to the role played by temperature to regulate the LRS/HRS states of VO$_{2}$ volatile memristors, it is expected that external temperature $T_{0}$, as provided by an external heater or the ambient, modulates the behavior of both VO$_{2}$ device and oscillator. Consequently, the external temperature will also impact ONN dynamics, affecting ONNs as analog computing architecture. For instance, the process of finding the ground-state of Ising machines can be implemented by gradually lowering the effective temperature (annealing)^24,25^, where such ground-state corresponds to the solution of optimization problems. Recently, an Ising Hamiltonian solver based on VO$_{2}$ oscillators has been demonstrated^26^.

In this work, we assess the role of the external temperature over the electrical behavior of VO$_{2}$ volatile memristors and oscillators. To this aim, we use technology Computer-Aided Design (TCAD) technique to run multi-physics simulations of VO$_{2}$ devices and oscillators. Physics-based multi-scale simulations are essential tools for investigating beyond CMOS devices, where the control of materials growth and micro/nanofabrication procedures is still to be fully mastered. Given the variability of nominally identical devices, the abstraction of trends in device behavior through experiments is quite challenging. On the contrary, physics-based simulations can precisely consider material variability and/or device geometry, which captures correlations and provides insights into device behavior. We perform 3D TCAD electrothermal simulations of VO$_{2}$ volatile memristors to accurately take into account Joule effect, heat dissipation and $T_{0}$. We show the dependence of *I* vs. $V_{\mathrm{D}}$ curves from $T_{0}$, and in particular, of the points of occurrence of resistive switching and its resetting. We are able to provide a simplified model for the evolution of such points with $T_{0}$. This yields the effect of modulation with $T_{0}$ of associated NDR region. We assess the impact of such modulation over VO$_{2}$ oscillator, considering that the load-line of oscillatory circuit does not depend on $T_{0}$. It is important to observe that the sensitivity of VO$_{2}$ oscillator to $T_{0}$ has a number of consequences. It can bring to cross-talk effects 1) self-induced, due to the local thermal build-up of VO$_{2}$ oscillator, or 2) among nearby VO$_{2}$ oscillators during ONN operativity. However, it also *de facto* embeds into VO$_{2}$ neuron-mimicking devices a (thermal) sensory function. Thus, our work provides essential TCAD tools on one hand to ascertain the thermal cross-talk effects in VO$_{2}$ oscillators, and on the other to engineer the response characteristics of VO$_{2}$ sensory neuron devices. Finally, we determine how figures of merit of VO$_{2}$ oscillator, such as frequency, voltage amplitude and average power per cycle, vary according to $T_{0}$. Our findings shed light on the interlinked thermal and electrical behavior of VO$_{2}$ oscillators that are helpful for providing guidelines for the implementation of ONN technology.

## TCAD electrothermal simulations of VO$_2$ crossbar device

We perform 3D TCAD electrothermal simulations of VO$_{2}$ crossbar (CB) devices fabricated on a silicon platform. The experimental VO$_{2}$ device is realized on a silicon substrate covered by 1 $\mu$m-thick SiO$_{2}$ layer^27^. The bottom Pt electrode is deposited after etching a trench in SiO$_{2}$ layer. Then, the 80-nm thick VO$_{2}$ layer is grown and subsequently shaped into a square of 5 $\mu$m-side. Finally, the top electrode is laid down to complete the CB geometry (Fig. 2b). Both electrodes have a width of 250 nm.

### Modeling of the VO$_{2}$ resistive switching

We use Silvaco Victory Mesh^28^ for 3D mesh rendering of the actual geometry of VO$_{2}$ device (Fig. 2b). This allows to emulate accurately 3D effects of thermal dissipation and the real geometry of current flow. Experimental VO$_{2}$ is a policrystalline, granular film due to the growth on SiO$_{2}$ substrate, with average size of grains of $\approxeq$ 50 nm^27^. However, in simulations we treat VO$_{2}$ as homogeneous material, because the active device region (about where the two contacts overlap) has a cross-section area of 250 nm by 250 nm, which amounts to a few grains. Thus, to consider the device “channel” as uniform is a good approximation that helps to reduce the computational time and complexity. We perform 3D TCAD electrothermal simulations of VO$_{2}$ volatile memristor by Silvaco Victory Device^29^. We model the VO$_{2}$ as a conductor material whose resistivity vs. temperature is described by a user-defined version of the Phase-Change Material (PCM) model^30,31^ (Fig. 2a). In static simulations, the resistivity ${\hat{\rho }}$ is linear with the temperature *T*, with different slopes $m_{\mathrm{LT}}$, $m_\mathrm{HT}$ depending on VO$_{2}$ being insulating ($T < T_{\mathrm{LT}}$) or metallic ($T > T_{\mathrm{HT}}$), respectively. ${\hat{\rho }}(T)$ is also linear across the IMT/MIT region ($T_{\mathrm{LT}}< T < T_{\mathrm{HT}}$). Because experimental curves of resistance vs. temperature show hysteresis (Fig. 2a), two different sets of parameters are used for the heating/cooling cycle. In time-dependent simulations, there will be a time lag for a sufficiently fast temperature change for the phase change to occur. Therefore, the time-dependent resistivity is modeled as:

1 $$\begin{aligned} \rho (t + \Delta t) = \rho (t) + ({\hat{\rho }}(T) - \rho (t)) \times \left[ 1 - \mathrm exp \left( - \frac{\Delta t}{\tau }\right) \right] \end{aligned}$$

where $\rho (t)$ is the resistivity at previous time step *t*, $\rho (t + \Delta t)$ is the resistivity at new time step and $\tau$ is a lifetime. The model also takes into account incomplete heating/cooling cycles, when they are reversed before achieving the full transition to the metal (or insulator) state. For example, if a cooling cycle starts at temperature $T_{B}$ such that $T_{\mathrm{LT, H}}< T_{B} < T_{\mathrm{HT, H}}$, before the heating cycle is completed, then the resistivity remains constant until the temperature $T_{A}$ is reached such that $\rho (T_{A})$ belongs to the cooling branch (Fig. 2a). This reproduces recent experimental findings^32^ on the behavior of VO$_{2}$ resistivity vs. temperature.

We calibrate the static PCM resistivity ${\hat{\rho }}_{\mathrm{PCM}}$ against experimental data of resistance $R_{\mathrm{exp}}$ vs temperature *T*^31^. Given the CB geometry, the cross-section of the current flow is not limited just to the region of the overlap between the top and bottom electrodes^31^, thus we cannot derive directly ${\hat{\rho }}$ from $R_{\mathrm{exp}} \times A_{\mathrm{C}} / L$, with *L* the VO$_{2}$ thickness and $A_{\mathrm{C}}$ the cross-section area of overlapping contacts. However, most of the current is indeed flowing therein^31^, thus, we simply assume that

2 $$\begin{aligned} {\hat{\rho }}_{\mathrm{PCM}} = R_{\mathrm{exp}} \times A_{\mathrm{Ceff}} / L \end{aligned}$$

for $A_{\mathrm{Ceff}} = A_{\mathrm{C}} \times a$, *a* being a parameter. Because VO$_{2}$ is modeled as a conductor, the conduction equation is solved as $J = E / \rho = - \nabla \phi / \rho$, where *J* is the current density, *E* is the electric field and $\phi$ is the electric potential. The Poisson equation and the heat flow equation

3 $$\begin{aligned} C \frac{\partial T_{\mathrm{L}}}{\partial t} = \nabla (K \nabla T_\mathrm{L}) + \frac{(\nabla \phi )^{2}}{\rho } \end{aligned}$$

are solved coupled self-consistently using the Finite Element Method (FEM). In Eq. (3) $T_{\mathrm{L}}$ is the local lattice temperature, *K* is the thermal conductivity and *C* is the heat capacity per unit volume. The Joule effect is accounted through the heat generation rate $\partial Q/ \partial t = \rho J^{2} = (\nabla \phi )^{2} / \rho$, where *Q* is the heat contributed by Joule effect. The VO$_{2}$ temperature is obtained by TCAD simulations through solution of Eq. (3). We use experimental current *I* vs. voltage across the VO$_{2}$ ($V_{\mathrm{D}}$) characteristics as a benchmark to validate our approach^31^. The experimental *I* - $V_{\mathrm{D}}$ data have been collected at the external temperature of $T_{0} = 303$ K as measured at the oxide surface close to the CB device (Fig. SI0). We emulate the experimental condition by setting a temperature of $T_{0} = 303$ K at the bottom surface of the VO$_{2}$ layer that we consider to be the thermal contact of TCAD simulations. We use a value of 0.06 W/(cm K)^33^ for the thermal conductivity of VO$_{2}$, and of 3 J/(cm$^{3}$ K)^34^ for its heat capacity per unit volume.

We obtain a good agreement with the experimental data^31^ (Fig. SI1) provided that 1) we set $a = 2.7$ in Eq. (2). Additionally, 2) we need to calibrate the interface thermal resistance of thermal contact set at the bottom of the device to achieve the same values of voltage $V_{\mathrm{IMT}}$ and current $I_{\mathrm{IMT}}$ at which the resistive switching occurs in simulated and experimental *I* - $V_{\mathrm{D}}$ curves. The interface thermal resistance of thermal contact accounts for heat dissipation through VO$_{2}$ / substrate interface, and it depends from material properties of VO$_{2}$ and SiO$_{2}$, and also from interface roughness, for example. Such interface thermal resistance is set as thermal conductance of thermal contact, which together with $T_{0}$ represent the thermal boundary condition. For simplicity sake, we consider the VO$_{2}$ / substrate interface as the main source of heat dissipation given that it is reasonable to assume that the heat dissipation across VO$_{2}$ / air interface, or VO$_{2}$ / top contact interface, can be, in comparison, neglected.

It is important to highlight that we do not fit VO$_{2}$ material parameters to match *I* - $V_{\mathrm{D}}$ curves, but we use physical meaningful values. This demonstrates that, overall, we are able to reproduce by simulations the salient features of the electrical behavior of VO$_{2}$ CB devices because we capture the physical mechanisms operating inside the device.

## Evolution of NDR region with external temperature

**Figure 3:**
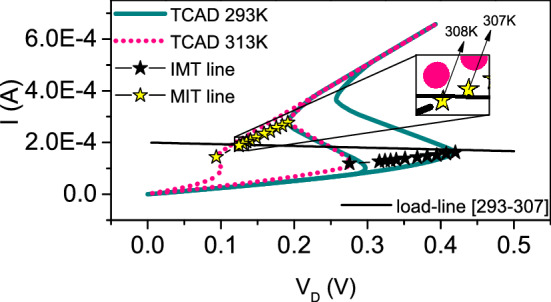
Simulated *I*–$V_{D}$ at external temperature of $T_{0} = 293$ K (solid dark cyan line) and $T_{0} = 313$ K (pink dotted line). The IMT/MIT points for $T_{0}$ ranging from 293 K (the rightmost ones) to 313 K (the leftmost ones) are shown as black (IMT) and yellow (MIT) starred lines, respectively. The black line is the load line valid for the temperature interval [293–307] K. The inset shows the MIT points for $T_{0} = 307$ K (on the right) and $T_{0} = 308$ K (on the left). It is evident that the MIT point for $T_{0} = 307$ K is above the load-line, while the MIT point for $T_{0} = 308$ K is below it.

We perform 3D TCAD electrothermal simulations of VO$_{2}$ crossbar (CB) devices at different external temperatures $T_{0}$. The simulated *I* - $V_{\mathrm{D}}$ curves for $T_{0} = 293$ K (dark cyan solid line) and $T_{0} = 313$ K (pink dotted line) are plotted in Fig. 3, showing the modulation effect of $T_{0}$. In particular, the values of voltage and current at which the resistive switching ($V_{\mathrm{IMT}}$, $I_{\mathrm{IMT}}$) and its reset ($V_\mathrm{MIT}$, $I_{\mathrm{MIT}}$) occur decrease with $T_{0}$. This can be easily interpreted: by increasing the background temperature, less and less heat has to be generated by Joule effect to achieve the temperature for resistive switching inside the volatile memristor. We denote ($V_{\mathrm{IMT}}$, $I_{\mathrm{IMT}}$) as IMT point and ($V_\mathrm{MIT}$, $I_{\mathrm{MIT}}$) as MIT point. The black (IMT) and yellow (MIT) starred lines of Fig. 3 are the TCAD simulated IMT/MIT points for $T_{0}$ ranging from 293 K to 313 K. The IMT and MIT points define the edges of the NDR region, which then, varies with $T_{0}$. The NDR region defines the operative regime of the oscillator circuit (Fig. 2d): it yields electrical oscillations only if the circuit load-line $I_{L} = (V_{\mathrm{DD}} - V) / R_{\mathrm{ext}}$ crosses it.

**Figure 4:**
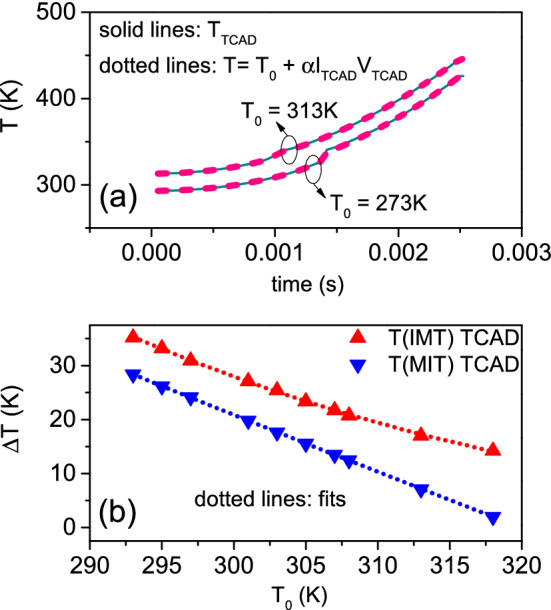
(**a**) Local temperatures probed in the geometrical center of the device as extracted by TCAD simulations (dark cyan solid lines) for $T_{0}$ equal to 293 and 313 K. We achieve a very good agreement with temperatures calculated after Eq. (4) for $\alpha = 5.15 \times 10^{5}$ K$/$W (pink dashed lines). (**b**) $\Delta T_{\mathrm{IMT}}$ (red triangles) and $\Delta T_\mathrm{MIT}$ (blue triangles) as determined by TCAD simulations for different $T_{0}$. The dotted lines are the correspondent fits by 2nd order polynomials.

**Figure 5:**
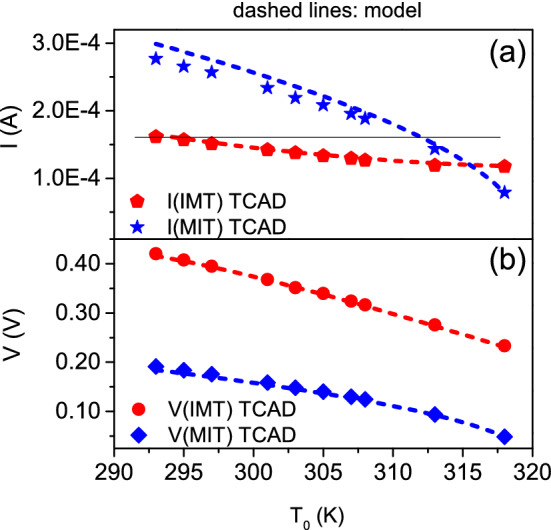
(**a**) $I_{\mathrm{IMT}}$ (red pentagons), $I_{\mathrm{MIT}}$ (blue stars) and (**b**) $V_{\mathrm{IMT}}$ (red circles), $V_{\mathrm{MIT}}$ (blue diamonds) as derived from TCAD simulations. The dashed lines are the currents and voltages as calculated from the proposed model. In (**a**), the black solid line is drawn parallel to $T_{0}$ axis. It intersects the $I_{\mathrm{IMT}}$ line at $T_{0} = 293$ K, and the $I_{\mathrm{MIT}}$ line at about $T_{0} = 312$ K.

We are able to model the dependence of IMT and MIT points with $T_{0}$ as obtained by 3D TCAD electrothermal simulations. Thus, we can predict the NDR region at any given $T_{0}$. In the following, we show the model for IMT points, the same applies to MIT points. We approximate the device as a homogeneous resistor, whose resistance is determined by $R = {\hat{\rho }}_{\mathrm{PCM}}(T_{ch}) \times L / A_\mathrm{Ceff}$. $T_{ch}$ is not the local temperature inside the device but a “global” temperature such that *R* is the device resistance at the given time. We assume $T_{ch}$ as the uniform temperature in the region of overlapping contacts if the current flux is confined therein. We correlate $T_{ch}$ to the device power as:

4 $$\begin{aligned} T_{ch} = T_{0} + \alpha \times I \times V \end{aligned}$$

where the electric power is converted into heat energy that dissipates to the ambient obeying Newton’s law of cooling. This corresponds to describing the heating effects in VO$_{2}$ with the same theory used for thermistors^35^. The validity of such approach has been experimentally demonstrated in the case of planar devices^19^. $\alpha$ is $(A \times {\overline{h}})^{-1}$, for *A* the heat transfer surface area, ${\overline{h}}$ the heat transfer coefficient averaged over *A*^36^, and it is related to how the VO$_{2}$ channel exchanges heat with the ambient, thus it depends on material properties and device geometry^19^. We consider that TCAD simulated temperature probed in the geometrical center of the device is a good approximation of $T_{ch}$, because most of the current flow is concentrated in the region of overlapping contacts^31^. Thus, we match the TCAD temperature to Eq. (4). We achieve a very good agreement by setting $\alpha = 5.15 \times 10^{5}$ K/W. Figure 4a shows the TCAD (solid lines) and the calculated temperatures (dashed lines) for $T_{0}$ equal to 293 and 313 K. Now, $T_{\mathrm{IMT}} = T_{0} + \alpha \times I_{\mathrm{IMT}} \times V_{\mathrm{IMT}} = T_{0} + \Delta T_{\mathrm{IMT}}$ is the temperature of resistive switching. It is noteworthy to observe that $T_{\mathrm{IMT}}$ depends on $V_{\mathrm{IMT}}$ and $I_{\mathrm{IMT}}$. In turn, $V_{\mathrm{IMT}}$ depends from $T_{\mathrm{IMT}}$, as well as $I_{\mathrm{IMT}}$ depends from $T_{\mathrm{IMT}}$. This means that there is not a closed-form expression of the relationship between $T_{\mathrm{IMT}}$ and $V_{\mathrm{IMT}}$, $I_{\mathrm{IMT}}$. We use TCAD simulation to derive $T_{\mathrm{IMT}}$ at different $T_{0}$. $\Delta T_{\mathrm{IMT}}$ vs $T_{0}$ is plotted in Fig. 4b (red triangles). We got an excellent match by fitting these points with a 2nd-order polynomial (red dotted line). We infer $R(T_{\mathrm{IMT}})$ from PCM model as $R(T_\mathrm{IMT}) = \rho (T_{\mathrm{IMT}}) \times L / A_{\mathrm{Ceff}}$. Finally, $I_{\mathrm{IMT}} = V_{\mathrm{IMT}} / R(T_{\mathrm{IMT}})$. However, from Eq. (4), $I_{\mathrm{IMT}} = \Delta T_{\mathrm{IMT}} / (\alpha \times V_{\mathrm{IMT}})$. By combining the two above equations together, we determine

5 $$\begin{aligned} V_{\mathrm{IMT}} = \sqrt{\frac{\Delta T_{\mathrm{IMT}} \times R(T_\mathrm{IMT})}{\alpha }} \end{aligned}$$

and we get $I_{\mathrm{IMT}}$ from $V_{\mathrm{IMT}}$ and $R(T_{\mathrm{IMT}})$. In Fig. 5a,b are plotted $I_{\mathrm{IMT}}$ and $V_{\mathrm{IMT}}$ against $T_{0}$, respectively, as extracted from TCAD simulations (red symbols) and as determined by our model (dashed red lines). We apply the same procedure also to $I_{\mathrm{MIT}}$ and $V_{\mathrm{MIT}}$ vs. $T_{0}$. The excellent agreement between simulated and calculated points in all the cases demonstrates that the behavior of IMT/MIT points with $T_{0}$ is correlated to the dependence of $T_\mathrm{IMT}$, $T_{\mathrm{MIT}}$ from $T_{0}$, expressed by Eq. (4). Thus, at least for the resistive switching and its resetting, the local in-homogeneity in resistivity as induced by the local temperature distribution can be ignored. The device can be reduced to a homogeneous resistor, whose resistance is determined by the temperature of the center of the device.

## Behavior of VO$_{2}$ oscillator with external temperature

VO$_{2}$ oscillator (Fig. 2d) is expected to depend as well as on $T_{0}$. For instance, figures of merit of the oscillator, such as the amplitude and period of the electrical signal at the output node, depend on $V_{\mathrm{IMT}}$ and $V_{\mathrm{MIT}}$, and then from $T_{0}$. This can be easily understood in the following way. In first approximation, the triggering thermal mechanism induced by Joule effect can be ignored, and VO$_{2}$ volatile memristor is just described as a two-state (LRS and HRS) variable resistor, activated by threshold voltages $V_{\mathrm{IMT}}$ and $V_{\mathrm{MIT}}$, respectively. Either LRS/HRS, the oscillator circuit equation is expressed by Kirchhoff’s law: $C_{\mathrm{ext}} \frac{dV}{dt} = I_{L} - I$^30,37^. At $V_{\mathrm{IMT}}$, the VO$_{2}$ is switched on (LRS) and the external capacitor $C_\mathrm{ext}$ charges, with the voltage at the output node $V_{\mathrm{out}}$ varying from $V_{\mathrm{L}} = V_{\mathrm{DD}} - V_{\mathrm{IMT}}$ to $V_{\mathrm{H}} = V_{\mathrm{DD}} - V_{\mathrm{MIT}}$. At $V_{\mathrm{MIT}}$, the VO$_{2}$ is switched off (HRS) and $C_{\mathrm{ext}}$ discharges, with $V_{\mathrm{out}}$ varying from $V_{\mathrm{H}}$ to $V_{\mathrm{L}}$. The cycles of capacitor charging and discharging are repeated periodically and yield the voltage oscillation. Then, the amplitude of voltage oscillations is $V_{\mathrm{H}} - V_{\mathrm{L}} = V_{\mathrm{IMT}}$ - $V_{\mathrm{MIT}}$. Moreover, the times for capacitor charging $\tau _{\mathrm{ch}}$ and discharging $\tau _{\mathrm{dis}}$ both depend on $V_{\mathrm{IMT}}$ and $V_{\mathrm{MIT}}$, as well as the oscillation period $\tau = \tau _{\mathrm{ch}} + \tau _\mathrm{dis}$^37,38^. We investigate by 3D TCAD electrothermal simulation the behavior of VO$_{2}$ oscillator with $T_{0}$. It is worthwhile to remember that I) IMT/MIT points vary with $T_{0}$, while II) the circuit parameters, that is the load-line $I_{L} = (V_{\mathrm{DD}} - V) / R_{\mathrm{ext}}$, do not depend from $T_{0}$. This produces a number of consequences.

- There is a finite range of external temperatures for which a fixed load-line crosses the associated NDR regions. That is, there exists a finite range of $T_{0}$ within which the VO$_{2}$ oscillator can oscillate.

**Figure 6:**
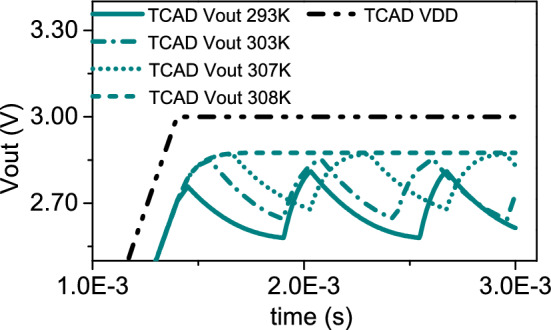
TCAD simulated voltage oscillations for $V_{\mathrm{DD}} = 3$ V, $R_{\mathrm{ext}} = 15$ k$\Omega$ and $C_{\mathrm{ext}} = 1.5 \times 10^{-7}$ F, at external temperature of $T_{0} = 293$ K (solid line), $T_{0} = 303$ K (dash-dotted line), $T_{0} = 307$ K (dotted line) and $T_{0} = 308$ K (dashed line).

For instance, in Fig. 3 is plotted the load-line (black solid line) corresponding to circuit parameters $V_{\mathrm{DD}} = 3$ V and $R_{\mathrm{ext}} = 15$ k$\Omega$. It 1) passes above all the IMT points for $T_{0} > 293$ K, and 2) is above MIT point at $T_{0} = 308$ K and below MIT point at $T_{0} = 307$ K (inset of Fig. 3). This means the load-line crosses the NDR regions in the interval of external temperatures [293–307] K. Figure 6 shows the simulated $V_{\mathrm{out}}$ for the above load-line ($C_\mathrm{ext} = 150$ nF) at $T_{0} = 293$ K (dark cyan solid line), $T_{0} = 303$ K (dark cyan dashed dotted line), $T_{0} = 307$ K (dark cyan dotted line) and $T_{0} = 308$ K (dark cyan dashed line). As expected, the oscillatory behavior is present for all the $T_{0}$ except 308 K. It also follows that there will be intervals of external temperatures for which it is impossible to find a common load-line such that it crosses all the correspondent NDRs. For instance, in Fig. 5a, the (black solid) line is drawn parallel to $T_{0}$ axis, which intercepts the $I_{\mathrm{IMT}}$ line at $T_{0} = 293$ K and the $I_{\mathrm{MIT}}$ line at about $T_{0} = 312$ K. This means that the NDR regions for $T_{0} > 312$ K cannot overlap with NDR region at $T_{0} = 293$ K. Thus, it does not exist a load-line which crosses the NDR regions from $T_{0} = 293$ K to above 312 K.

- There are temperatures for which the NDR region does not exist.

For example, in Fig. 5a the $I_{\mathrm{IMT}}$ line crosses the $I_{\mathrm{MIT}}$ line at about $T_{0} = 315$ K. This means that for $T_{0} > 315$ K the NDR region does not exist because $I_{\mathrm{MIT}} < I_{\mathrm{IMT}}$.

**Figure 7:**
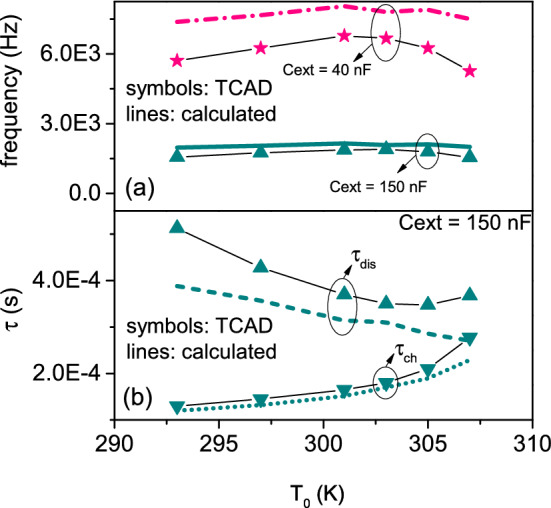
(**a**) Frequency of voltage oscillations vs the external temperature for $C_{\mathrm{ext}} = 150$ nF (dark cyan triangles) and $C_{\mathrm{ext}} = 40$ nF (pink stars). The curves obtained by the model of capacitor charging and discharging^37^ (pink dashed and dark cyan solid lines) are also plotted. (**b**) TCAD simulated periods of charging $\tau _{\mathrm{ch}}$ (downward triangles) and discharging $\tau _{\mathrm{dis}}$ (upward triangles) for $C_\mathrm{ext} = 150$ nF. The dotted and dashed lines are the curves for $\tau _{\mathrm{ch}}$, $\tau _{\mathrm{dis}}$ by applying the circuit equations^37^.

We perform TCAD electrothermal simulations of VO$_{2}$ oscillator at different external temperatures $T_{0}$. The oscillator circuit is simulated by considering the external resistance $R_{\mathrm{ext}}$ and capacitance $C_{\mathrm{ext}}$ as lumped element boundaries of VO$_{2}$ memristor. Any intrinsic device capacitance is accurately accounted into TCAD simulation of device electrostatics. We extract from simulated $V_{\mathrm{out}}$ the frequency against $T_{0}$ in interval [293–307] K, for the load-line correspondent to $V_{\mathrm{DD}} = 3$ V, $R_{\mathrm{ext}} = 15$ k$\Omega$. The results for $C_{\mathrm{ext}} = 150$ nF (dark cyan lines) and $C_{\mathrm{ext}} = 40$ nF (pink lines) are plotted in Fig. 7a. It can be appreciated the change of oscillator frequencies induced by variation of external temperature in the range [293–307] K. In particular, the frequency increases with $T_{0}$, it achieves a maximum, and then it decreases again at higher $T_{0}$. This behavior applies to all the probed values of the external capacitance. The decrease of frequency at $T_{0} = 293$ K and $T_{0} = 307$ K is easily interpreted with the fact that the load-line gets close to IMT point at $T_{0} = 293$ K and to MIT point at $T_{0} = 307$ K. Then, at these two temperatures, $I_{L} - I$ is very small. This, in turn, slows down the variation of voltage with time, as determined by Kirchhoff’s law. This is also coherent with the fact that at $T_{0} < 293$ K and $T_{0} > 307$ K the load-line does not anymore cross the associated NDR regions. Thus, there is no more oscillatory behavior, which means zero frequency. We plot in Fig. 7b the periods of capacitor charging $\tau _{\mathrm{ch}}$ (downward triangles) and discharging $\tau _{\mathrm{dis}}$ (upward triangles) vs $T_{0}$ ([293–307] K). Their sum yields the oscillator period $\tau$. Figure SI2 of Supporting Information (SI) shows the correspondent curves for $C_{\mathrm{ext}} = 40$ nF. Overall, TCAD simulated $\tau _{\mathrm{ch}}$ and $\tau _{\mathrm{dis}}$ have opposite trends with $T_{0}$ (belonging to [293–307] K): of increasing for $\tau _{\mathrm{ch}}$ and of decreasing for $\tau _{\mathrm{dis}}$. The percentage of variation between maximum and minimum values compared to the minimum one for TCAD simulated $\tau _{\mathrm{ch}}$ varies between 113% ($C_{\mathrm{ext}} = 150$ nF) and 87% ($C_{\mathrm{ext}} = 40$ nF), while for TCAD simulated $\tau _{\mathrm{dis}}$ it is comprised between 46% ($C_{\mathrm{ext}} = 150$ nF) and 35% ($C_{\mathrm{ext}} = 40$ nF). The percentage of variation between maximum and minimum values compared to the minimum one for TCAD simulated frequency is between 22% ($C_{\mathrm{ext}} = 150$ nF) and 29% ($C_{\mathrm{ext}} = 40$ nF). The decreased variability of frequency with $T_{0}$ is explained with the partially compensating effects of the opposed trends of $\tau _{\mathrm{ch}}$ and $\tau _{\mathrm{dis}}$.

We complete the survey of effects induced by $T_{0}$ over the VO$_{2}$ oscillator. We derive the variation of voltage amplitude (Fig. SI6a of SI) and average power per cycle (Fig. SI6b of SI) against the external temperature (range [293–307] K) for the fixed load-line $V_{\mathrm{DD}} = 3$ V, $R_{\mathrm{ext}} = 15$ k$\Omega$. $C_{\mathrm{ext}}$ ranges from 150 nF to 40 nF. The voltage amplitude (Fig. SI6a) decreases with the external temperature, due to the trend of $V_{\mathrm{IMT}}$ and $V_{\mathrm{MIT}}$ with $T_{0}$ (Fig. 5b). As expected, there is no dependence from $C_\mathrm{ext}$. Finally, we determine the average power per cycle $< P>$. We find that it lies in the range of values from $5.1 \times 10^{-4}$ W to $5.7 \times 10^{-4}$ W within the temperature interval [293 – 307] K and for all the external capacitance values.

## Conclusions

In this paper, we investigate the effect of ambient temperature over VO$_{2}$ volatile memristors and oscillators, using TCAD multi-physics simulations. We perform 3D TCAD electrothermal simulations of VO$_{2}$ volatile memristors to accurately take into account Joule effect, heat dissipation and $T_{0}$. Since our simulations link in a physical way VO$_{2}$ material properties (for example: resistivity vs temperature relationship) and oscillator circuit parameters (load-line), we are able to extract a number of consequences. The most important is to predict and explain the variation of oscillator frequency with the external temperature $T_{0}$. In particular, the variation of frequency is due to 1) the dynamics of variation of NDR region induced by $T_{0}$, and, on the contrary, 2) the stability against $T_{0}$ of load-line. It is important to observe that this behavior has been recently experimentally demonstrated for VO$_{2}$ memristors in self-oscillatory regime^39^. This modulation effect of $T_{0}$ over behavior of VO$_{2}$ oscillators has multiple repercussions. It can bring to cross-talk effects (1) self-induced, due to the local thermal build-up of VO$_{2}$ oscillator, or (2) among nearby VO$_{2}$ oscillators during ONN operativity. In this last case, the amount of cross-talk will depend on a number of factors, among them the distance among oscillators, and thus it may ultimately set a limit to integration of VO$_{2}$ oscillators. Finally, the sensitivity of VO$_{2}$ oscillator to $T_{0}$ also *de facto* embeds into VO$_{2}$ neuron-mimicking device a (thermal) sensory function. Thus, our work provides essential TCAD tools on one hand to ascertain the thermal cross-talk effects in VO$_{2}$ oscillators, and on the other to engineer the response characteristics of VO$_{2}$ sensory neuron devices.

## Supplementary Information

Supplementary Figures.

## Data Availability

The datasets used and/or analyzed during the current study available from the corresponding author on reasonable request.
