# Supplementary material for: Role of ambient temperature in modulation of behavior of vanadium dioxide volatile memristors and oscillators for neuromorphic applications
Source: Sci Rep. 2022 Nov 12;12:19377. doi: 10.1038/s41598-022-23629-4 (PMC9653463; doi:10.1038/s41598-022-23629-4)
Supplement: Supplementary file 1 — Supplementary Figures. [file 41598_2022_23629_MOESM1_ESM.pdf]

Supporting Information

Role of Ambient Temperature in Modulation of Behavior of Vanadium Dioxide Volatile Memristors and Oscillators for Neuromorphic Applications

Stefania Carapezzi, [1] Corentin Delacour [1], Andrew Plews [3], Ahmed Nejim [3], Siegfried Karg [4], Aida Todri-Sania * [1,2]*

[1]Microelectronics Department, LIRMM, University of Montpellier, CNRS, Montpellier, 34095, France

[2]Department of Electrical Engineering, Eindhoven University of Technology, Eindhoven, 5612 AP, Netherlands

[3]Silvaco Europe Ltd., St Ives, PE27 5JL, United Kingdom.

[4]Department of Science and Technology, IBM Research Europe - Zurich, Ruschlikon, 8803, Switzerland

* Address correspondence to stefania.carapezzi@gmail.com and aida.todri@lirmm.fr

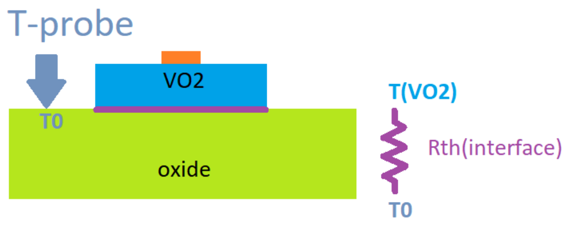

Fig. SI0 Experimental set-up for measurement of external temperature T_0.

The experimental I-V curve of VO_2 volatile memristor has been taken at the external temperature of T_0 = 303 K as measured at the oxide surface close to the CB device. Fig. SI0 reproduces these experimental conditions. It is a reasonable approximation to assume that T_0 is the temperature at the bottom of the device (thermal contact). In our simulations, we specified T_0 and the thermal conductance as thermal boundary condition at the thermal contact. The thermal conductance accounts for the interface thermal resistance of VO_2 / substrate interface, and it depends from material properties of VO_2 and SiO_2, and also from interface roughness, for example. The thermal conduction across VO_2 / substrate regulates the heat dissipation. In turn, it modulates the electrical output of VO_2 volatile memristor, as we investigated in reference [38] in the paper. It also enters into the process of thermal build-up around the VO_2 volatile memristor.

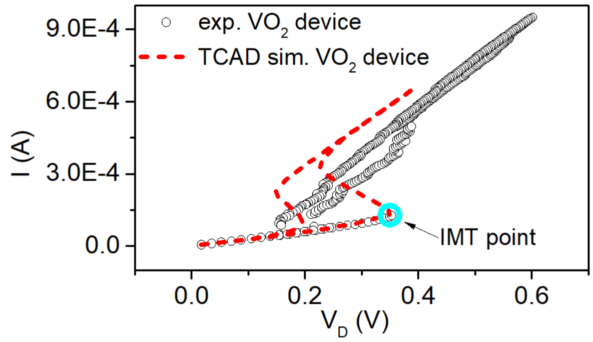

Fig. SI1 Experimental (symbols) and TCAD simulated (red dashed line) current I vs voltage across the VO_2 (V_D) curves. The experimental I vs. V_D were obtained in current-controlled mode. We perform I vs. V_D simulations in voltage-controlled mode with an external resistance of R_{ext} = 1 kΩ connected in series with the simulated VO_2 device, since it is easier to achieve convergence in this way. Both experimental and simulated curves agree very well before IMT point. However, when VO_2 is in metallic state, TCAD data slightly overestimates the experimental ones. This is likely related to the experimental switched region being smaller than the simulated region, which is about equal to the region where contacts overlap.

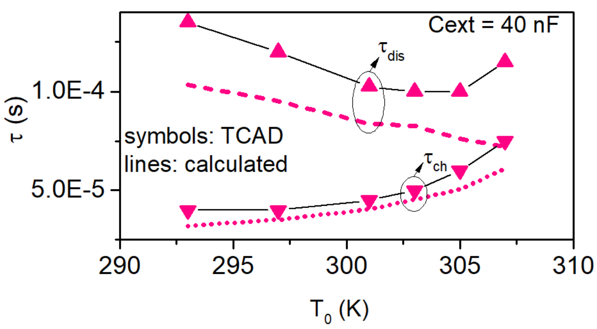

Fig. SI2 TCAD simulated periods of charging τ_{ch} (downward triangles) and discharging τ_{dis} (upward triangles) for C_{ext} = 40 nF. The dashed and dotted lines are the curves for τ_{ch}, τ_{dis} by applying the model of capacitor charging and discharging at threshold voltages V_{IMT} and V_{MIT} (reference [37] in the paper).

We apply the model of capacitor charging and discharging at threshold voltages V_{IMT} and V_{MIT} (reference [37] in the paper) to calculate τ_{ch} and τ_{dis}. These latters depend on 1) the load-lines parameters, 2) the resistance R_{ins} of insulator VO_2 and the resistance R_{met} of metallic VO_2, and 3) V_{IMT} and V_{MIT}. R_{ins}, R_{met}, V_{IMT}, V_{MIT} are estimated from TCAD simulations. We find R_{met} = 599 Ω for all T_0, while R_{ins} slightly varies with T_0, from 3584 kΩ (293 K) to 3112 kΩ (307 K). Fig. 7b in the manuscript (C_{ext} = 150 nF) and Fig. SI2 (C_{ext} = 40 nF) show the calculated τ_{ch} (dotted lines) and τ_{dis} (dashed lines). The trends with T_0 (range [293 -- 307] K) are similar to the ones for TCAD simulated values: of increasing for τ_{ch} and of decreasing for τ_{dis}.

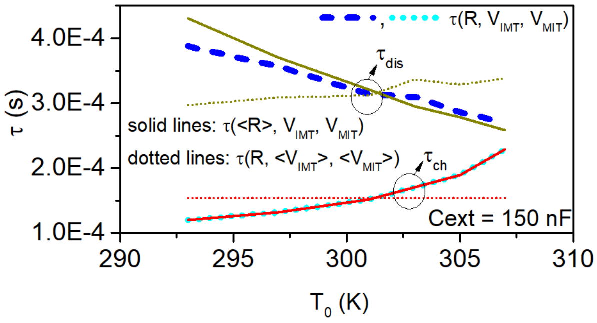

Fig. SI3

We repeat the calculation of τ_{ch} and τ_{dis} by considering A) averaged, fixed values of $<R_{ins}>$, $<R_{met}>$, while V_{IMT} and V_{MIT} are dependent on T_0, and B) averaged, fixed values of $<V_{IMT}>$ and $<V_{MIT}>$, while R_{ins}, R_{met} are dependent on T_0. The results (C_{ext} = 150 nF) are plotted in Fig. SI3 of SI. It can be seen that the trends of calculated τ_{ch} and τ_{dis} with all R_{ins}, R_{met}, V_{IMT}, V_{MIT} dependent on T_0 are matched almost exactly in case B), while the departure is evident in case A). This gives evidence that the behavior of V_{IMT} and V_{MIT} with T_0 plays the major role in determining the of τ_{ch} and τ_{dis} with T_0, and, in turn, the trend of calculated frequency.

We determine the percentage of departure of calculated τ_{ch}, τ_{dis} compared to simulated τ_{ch}, τ_{dis} (Fig. SI4 of SI). We find that the calculated values overestimate the simulated ones, slightly more for C_{ext} = 40 nF than for C_{ext} = 150 nF. For most values of T_0 the overestimation is about 10% for τ_{dis} and about 20% for τ_{ch}.

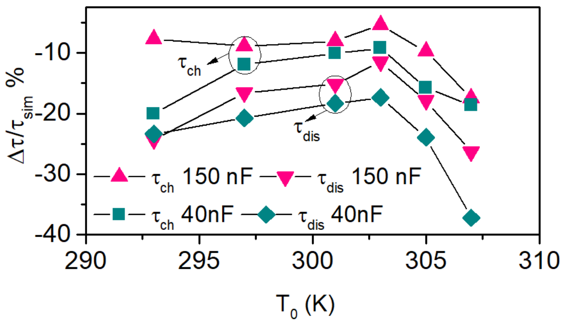

Fig. SI4 Percentage of departure of calculated τ_{ch}, τ_{dis} compared to simulated τ_{ch}, τ_{dis} for C_{ext} = 40 nF (dark cyan symbols) and for C_{ext} = 150 nF (pink symbols).

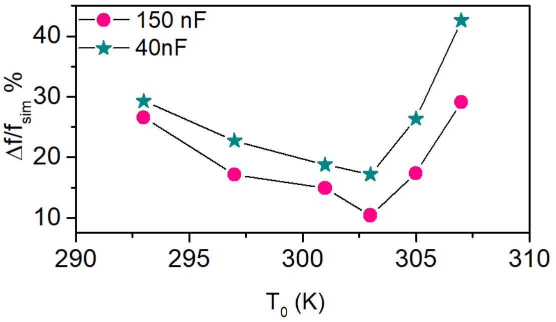

Fig. SI5 Percentage of departure of calculated frequency compared to simulated one for C_{ext} = 40 nF (dark cyan symbols) and for C_{ext} = 150 nF (pink symbols).

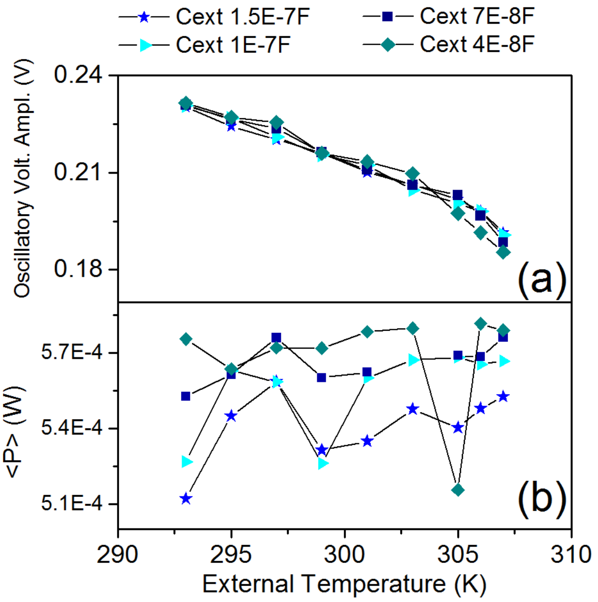

Fig. SI6 (a) Amplitude and (b) average power per cycle < P > of the voltage oscillations, for C_{ext} = 150 nF (blue stars), C_{ext} = 100 nF (cyan triangles), C_{ext} = 70 nF (dark blue squares) and C_{ext} = 40 nF (dark cyan diamonds).
